# Genome-wide identification of pathogenicity factors of the free-living amoeba *Naegleria fowleri*

**DOI:** 10.1186/1471-2164-15-496

**Published:** 2014-06-19

**Authors:** Denise C Zysset-Burri, Norbert Müller, Christian Beuret, Manfred Heller, Nadia Schürch, Bruno Gottstein, Matthias Wittwer

**Affiliations:** Biology Division, Spiez Laboratory, Federal Office for Civil Protection, Austrasse, Spiez, CH-3700 Switzerland; Institute of Parasitology, University of Bern, Länggassstrasse 122, CH-3012 Bern, Switzerland; Graduate School for Cellular and Biomedical Sciences, University of Bern, Freiestrasse 1, CH-3012 Bern, Switzerland; Mass Spectrometry and Proteomics, Department of Clinical Research, University Hospital, Bern, CH-3010 Switzerland

**Keywords:** Naegleria fowleri, Primary amoebic meningoencephalitis, Whole genome sequencing, RNA sequencing, Comparative proteomics, Pathogenicity factors

## Abstract

**Background:**

The free-living amoeba *Naegleria fowleri* is the causative agent of the rapidly progressing and typically fatal primary amoebic meningoencephalitis (PAM) in humans. Despite the devastating nature of this disease, which results in > 97% mortality, knowledge of the pathogenic mechanisms of the amoeba is incomplete. This work presents a comparative proteomic approach based on an experimental model in which the pathogenic potential of *N. fowleri* trophozoites is influenced by the compositions of different media.

**Results:**

As a scaffold for proteomic analysis, we sequenced the genome and transcriptome of *N. fowleri*. Since the sequence similarity of the recently published genome of *Naegleria gruberi* was far lower than the close taxonomic relationship of these species would suggest, a *de novo* sequencing approach was chosen. After excluding cell regulatory mechanisms originating from different media compositions, we identified 22 proteins with a potential role in the pathogenesis of PAM. Functional annotation of these proteins revealed, that the membrane is the major location where the amoeba exerts its pathogenic potential, possibly involving actin-dependent processes such as intracellular trafficking via vesicles.

**Conclusion:**

This study describes for the first time the 30 Mb-genome and the transcriptome sequence of *N. fowleri* and provides the basis for the further definition of effective intervention strategies against the rare but highly fatal form of amoebic meningoencephalitis.

**Electronic supplementary material:**

The online version of this article (doi:10.1186/1471-2164-15-496) contains supplementary material, which is available to authorized users.

## Background

*Naegleria* species are free-living amoebae found in soil and water throughout the world [1]. Although approximately 30 species have been recognized so far, *Naegleria fowleri* is the only human pathogen that causes primary amoebic meningoencephalitis (PAM) [2]. Infection occurs when water contaminated by *N. fowleri* enters the noses of swimmers and the amoebae reach the central nervous system through the olfactory nerve tract [3]. Several days after infection, patients suffer from severe inflammation of the brain and meninges, accompanied by headache, fever, vomiting, nausea and behavioral abnormalities. Because most infected individuals fail to be diagnosed rapidly, they die within one to two weeks after exposure to the infectious water source [3, 4]. The drug of choice for treating PAM is the antifungal drug amphotericin B. However, no more than a dozen patients out of approximately 350 reported PAM cases have been treated successfully with amphotericin B, either alone or in combination with other drugs [5–7]. Hence, *N. fowleri* is very problematic due to the rapid onset and destructive nature of the disease as well as the lack of effective treatments, rather than the number of cases worldwide.

Knowledge of the genome of *N. fowleri* is needed to provide insights into the pathogenetic mechanisms of the amoeba as a basis for developing more effective therapies as well as more rapid diagnostic tools. Here, we present an approach consisting of whole-genome sequencing in combination with proteomic analysis for identifying potential pathogenicity factors in *N. fowleri.* The genome of its non-pathogenic relative *Naegleria gruberi* has recently been sequenced [8]. A comparative analysis of the genomes of *N. gruberi* and *N. fowleri* based on a 60-kb nuclear segment showed less similarity between them than the present understanding of the phylogenetic relationships of *Naegleria* species would have led us to expect [9]. Therefore, the genome of *N. gruberi* is not suitable as a reference for genome assembly, and thus, a *de novo* sequencing approach had to be applied for determination of the complete genome sequence of *N. fowleri.* Furthermore, due to the substantial genetic differences observed, the application of a comparative genomic approach between pathogenic *N. fowleri* and non-pathogenic *N. gruberi* to define pathogenicity factors may be misleading. In the present work, we conducted an intra-species comparison of highly and weakly pathogenic *N. fowleri* trophozoites based on the model published by Burri *et al.* [10]. This model showed that *N. fowleri* trophozoites maintained in either Nelson’s medium or PYNFH medium supplemented with liver hydrolysate (LH, PYNFH/LH medium) are highly pathogenic in mice and demonstrate rapid *in vitro* proliferation, whereas trophozoites cultured in PYNFH medium are weakly pathogenic with a slower growth. Although the pathogenicity cannot be explained by different cytotoxicity mechanisms or by the presence of membrane vesicles in this model, it enables to investigate the pathogenesis of *N. fowleri* under defined experimental conditions [10].

The evaluation of sequencing data is a computationally challenging task due to the volume of data involved and because of statistical interference in the algorithms used for elucidating the genomic organization of novel eukaryotic genomes. The identification of protein coding regions in *de novo*-sequenced eukaryotic genomes based solely on *ab initio* computational algorithms is prone to specificity and sensitivity issues due to the lack of validated gene training sets. In this work, the obtained *in silico* gene-finding results were partially substantiated by experimental proteomic data. Furthermore, the search for potential pathogenicity factors was based on proteomic expression profiling of highly and weakly pathogenic *N. fowleri*, rather than, at least in this stage of research, less reliable transcriptomic data.

## Results

### Genomic DNA sequencing

As a pre-requisite for obtaining insight into the pathogenic mechanism of *N. fowleri*, the genome of the amoeba was sequenced using both Illumina HiSeq 2000 and Roche 454 GS FLX technology. The DNA isolated for whole-genome sequencing was composed of 2 times more plasmid DNA and 18 times less mitochondrial DNA than genomic DNA; thus, no substantial enrichment of either type of DNA was present in the starting material used for sequencing. In a first step, DNA was sequenced with the Illumina HiSeq 2000 platform, resulting in approximately 116 million 100-bp paired-end reads with an insert size of 300 bp. The *de novo* assembly of these short 100-bp reads was facilitated by 454 backbone sequencing, providing approximately 350,000 single reads with an average length of 378 bp. Finally, the assembly was improved with information from a mate-pair library composed of approximately 400 million Illumina reads with an insert size of 3 kb. In total, over 500 million reads were *de novo* assembled into 1,124 scaffolds with an average coverage of 770x and an N50 of 136,406. The nuclear genome of *N. fowleri* has a size of 29,619,856 bp and is AT-rich, with a GC content of only 35.4% (Tables 1 and 2). The calculation of the genome size via flow cytometry indicated that the *N. fowleri* genome is approximately 66 Mb. Based on the 29,619,856-bp size of the *de novo-*assembled genome, the genome of *N. fowleri* is considered diploid.

**Table 1:** **Summary of the** ***N. fowleri*** **genome**

Parameter	Number
Haploid genome size (bp)	29,619,856
Sequence contigs (bp)	4,339
N50 of contigs (bp)	17,724
Sequence scaffolds	1,124
N50 of scaffolds (bp)	136,406

**Table 2:** **Comparison of the** ***N. fowleri*** **with the** ***N. gruberi*** **genome**

Parameter	***N. fowleri***	***N. gruberi***
Haploid genome size (Mbp)	29.62	40.96
GC content (%)	35.4	33.1
Open reading frames	17,252	15,727
Bidirectional best BLAST hit	13,495	

### Transcriptome assembly and annotation

**Figure 1:**
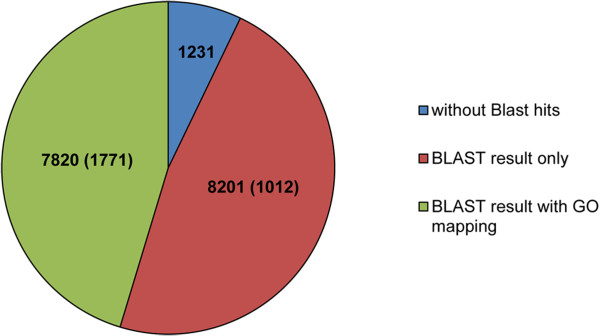
**Overview of the** ***N. fowleri*** **genome annotation.** From a total of 17,252 predicted open reading frames (ORFs), 1,231 remained without BLASTp hits, for 8,201 solely a BLASTp result was found and to 7,820 a Gene Ontology (GO) term could additionally assigned. The numbers in brackets indicate the number of ORFs that have been retrieved by proteomic analysis.

To generate a database for protein identification via nano-liquid chromatography tandem mass spectrometry (nano-LC MS/MS), RNA was sequenced using the Illumina HiSeq 2000 platform, resulting in approximately 229 million 100-bp paired-end reads. Using Trinity and the program cd-hit for redundancy filtering, 17,252 open reading frames (ORFs) were predicted (Table 2 and Figure 1).These ORFs were then used as a database for the identification of proteins in the 2D gel spots and 1D gel slices (see below). Only 0.2% of the predicted ORFs failed to align to the draft genome.The standalone BLASTp search against the RefSeq-protein database resulted in a significant hit for 16,021 of the 17,252 ORFs. To 7,820 of these 16,021 significant blast hits a Gene Ontology (GO) term could be assigned using the annotation pipeline Blast2GO (Figure 1).

### Genome similarity

**Figure 2:**
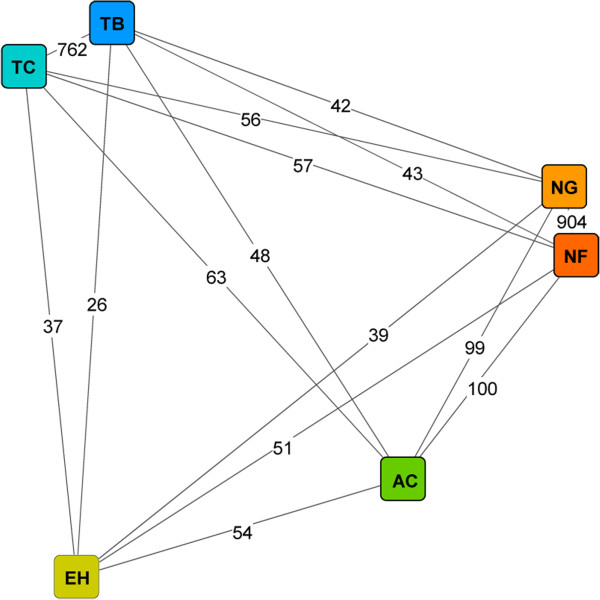
**Genome similarity network.** The genome similarity network was reconstructed using *N. fowleri* (NF) ORFs and EST sequences from *N. gruberi* (NG), *A. castellanii* (AC), *E. histolytica* (EH), *T. brucei* (TB) and *T. cruzi* (TC) using Evolutionary Gene and Genome Network (EGN) software. The graph was visualized using Cytoscape 3.0.1. The length of the edges is the inverse proportion of shared gene families. The number of shared gene families is indicated on the corresponding edges.

As the genome of *N. gruberi* was sequenced in 2010 [8], there is an important question regarding the relationship of *N. gruberi* to other *Naegleria* species, especially to its pathogenic relative *N. fowleri*. To resolve the degree of relationships between *N. gruberi* and *N. fowleri* at the molecular level*,* we reconstructed a genome similarity network based on EST sequences (Figure 2). As additional groups for comparison, two other amoeba, *Entamoeba histolytica* and *Acanthamoeba castellanii,* belonging to class Amoebozoa, and *Trypanosoma brucei* and *Trypanosoma cruzi,* belonging to class Euglenozoa, which is the most closely related group to the Heterolobosea (including all *Naegleria* species), were used. In this network, *N. fowleri* and *N. gruberi* share substantially more gene families than *N. fowleri* shares with the other species assessed, thus positioning *N. fowleri* as the closest relative to *N. gruberi*. Regarding the other analyzed amoeba, *N. fowleri* is closer to *A. castellanii* than to *E. histolytica*.

In an additional approach, BLASTn searches suggested low similarity between the coding sequences of *N. fowleri* and *N. gruberi*, as only 32.1% of the 17,252 predicted ORFs aligned to the *N. gruberi* genome (>99.0% of the ORFs matched the *de novo*-assembled *N. fowleri* genome). Despite the low similarity on nucleotide level, 78.2% of the *N. fowleri* ORFs showed a BLASTp hit with *N. gruberi* genes (Table 2).

### Identification of potential pathogenicity factors

**Figure 3:**
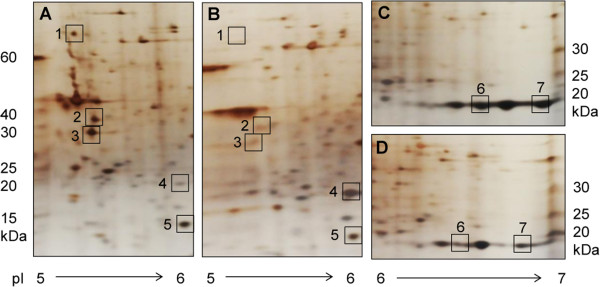
**Comparative 2D gel electrophoresis of highly (A and C) and weakly (B and D) pathogenic** ***N. fowleri.*** The proteomes of trophozoites were separated via 2D gel electrophoresis, and differentially expressed proteins (squares) were excised and identified via nano-liquid chromatography tandem mass spectrometry (nano-LC MS/MS). In the figure, only enlarged images from gel segments with differential spot patterns are shown. The numbers correspond to the identified proteins listed in Table 3. Spot 5 was used as a control, representing a protein (cofilin) with equivalent expression in highly and weakly pathogenic trophozoites.

The identification of pathogenicity factors in *N. fowleri* is an important step in revealing the mechanisms that are responsible for the destructive nature of PAM. Through comparative 2D gel electrophoresis, we obtained an overview of the proteomes of highly and weakly pathogenic *N. fowleri* (Figure 3)*.* For comparative visual quantification of the obtained protein concentrations, the actin-binding protein cofilin, which was expressed at equivalent levels under the two conditions, was used as a control protein spot. As seen in Figure 3 and Table 3, heat shock protein 70 (hsp70) [11], actin 1 and 2 [11–18] as well as the membrane protein Mp2CL5 [19], all of which are known potential pathogenicity factors, were expressed at increased levels in highly pathogenic compared to weakly pathogenic trophozoites. This observation confirmed the potential of all four proteins to contribute to the pathogenicity of *N. fowleri*. Moreover, we identified cyclophilin as being strongly overexpressed in highly pathogenic trophozoites, suggesting that this protein is an additional potential pathogenicity factor. Conversely, another protein, the hsp20 domain-containing protein, was over-expressed in weakly pathogenic *N. fowleri.*

**Table 3:** **Proteins that were differentially expressed in highly and weakly pathogenic** ***N. fowleri,*** **as identified via 2D gel electrophoresis in combination with nano-LC MS/MS**

Spot no	Accession	Protein description	Species	Mol. weight	Theoretical pI	Regulation
1	AY684788	Heat shock protein 70 (hsp70)	*N. fowleri*	71408 Da	5.14	up
2	M90311	Actin 1	*N. fowleri*	41728 Da	5.26	up
3	M90312	Actin 2	*N. fowleri*	41153 Da	5.23	up
4	XM_002673759	Hsp20 domain containing protein	*N. gruberi*	19836 Da	6.20	down
5	XM_002669269	Cofilin (control)	*N. gruberi*	15384 Da	5.90	equal
6	XM_002681214	Cyclophilin	*N. gruberi*	19333 Da	6.29	up
7	AY049749	Membrane protein Mp2CL5	*N. fowleri*	19932 Da	6.82	up

The molecular weight and theoretical pI values of the identified proteins were calculated according to the *Compute pI/Mw tool* from the SIB Bioinformatics Resource Portal (http://web.expasy.org/compute_pi/). The results of visual quantification of the protein concentrations found in highly pathogenic compared to weakly pathogenic trophozoites are indicated in the last column (Regulation).

**Figure 4:**
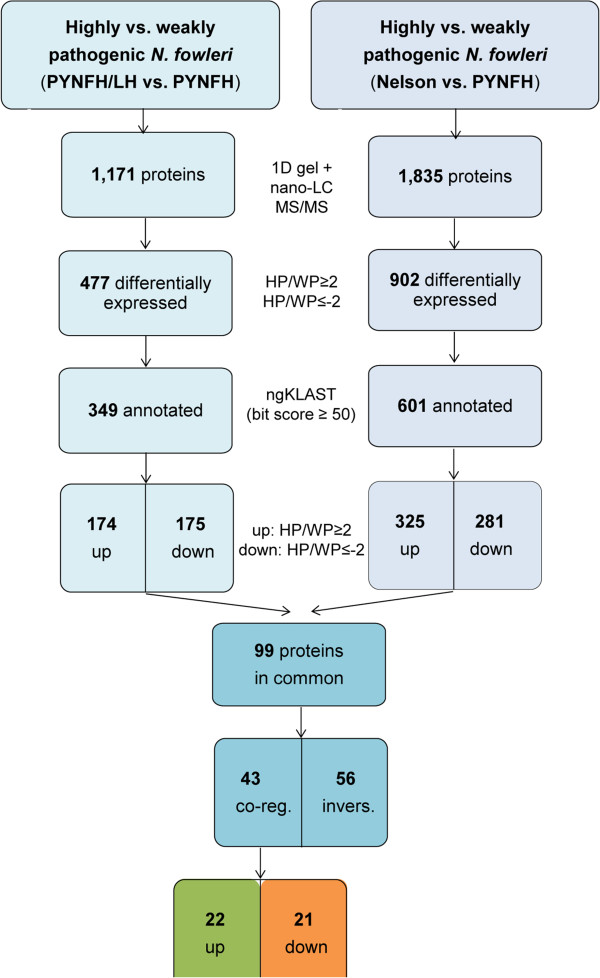
**Workflow: Identification of potential pathogenicity factors in** ***N. fowleri.*** By performing 1D gel electrophoresis in combination with nano-liquid chromatography tandem mass spectrometry (nano-LC MS/MS), a total of 1,171 proteins were found in comparison group 1, i.e., highly pathogenic (HP) trophozoites in PYNFH/LH medium versus weakly pathogenic (WP) trophozoites in PYNFH medium; in comparison group 2, i.e., highly pathogenic trophozoites in Nelson’s medium versus weakly pathogenic trophozoites in PYNFH medium, a total of 1,835 proteins were found. To identify proteins that were differentially expressed between highly and weakly pathogenic *N. fowleri*, a cut-off of a twofold change in expression (HP/WP ≥ 2 for up-regulated (up) and HP/WP ≤ −2 for down-regulated (down) proteins) was chosen. Annotation by the program ngKLAST (http://www.korilog.com) with a bit score equal to or greater than 50 resulted in 349 annotated proteins in comparison group 1 and 601 annotated proteins in comparison group 2. Among the 99 proteins found to be up- or down-regulated in both comparison groups, 43 proteins were co-regulated (co-reg.), while 56 proteins were inversely (invers.) regulated, i.e., up-regulated in one and down-regulated in the other comparison group. Among the 43 co-regulated proteins, the 22 components that were up-regulated in the highly pathogenic trophozoites were considered to be potential pathogenicity factors in *N. fowleri.*

In a second step, by performing 1D gel electrophoresis in combination with nano-LC MS/MS (Figure 4), we detected a total of 2,166 proteins, 477 of which were differentially expressed between weakly pathogenic trophozoites maintained in PYNFH medium and highly pathogenic trophozoites in PYNFH/LH medium (comparison group 1). Furthermore, 902 proteins were differentially expressed in weakly pathogenic trophozoites maintained in PYNFH medium and highly pathogenic trophozoites in Nelson’s medium (comparison group 2). Approximately two-thirds of the proteins showing different expression levels could be annotated by ngKLAST (see Methods). Using the protein expression level in weakly pathogenic *N. fowleri* as a reference, we observed 175 up- and 174 down-regulated proteins in trophozoites grown in PYNFH/LH as well as 281 up- and 325 down-regulated proteins in trophozoites grown in Nelson’s medium. Among these 950 differentially expressed and annotated proteins, only 99 were found to be up- or down-regulated in both highly pathogenic populations. Among these proteins, 43 were co-regulated in both highly pathogenic groups, while the remaining 56 were inversely regulated, i.e., up-regulated in one highly versus weakly pathogenic comparison group and down-regulated in the other comparison group. Among these 43 proteins that were co-regulated in both highly pathogenic *N. fowleri* populations, 22 components were up-regulated and were, thus, finally considered to represent potential pathogenicity factors.

**Figure 5:**
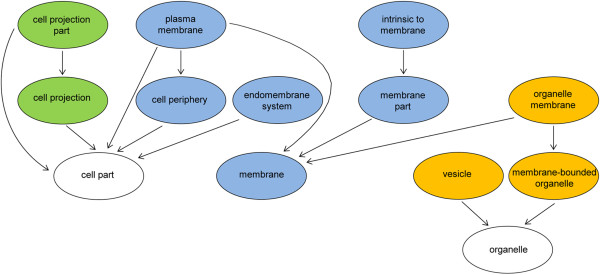
**Cellular component analysis of potential pathogenicity factors from** ***N. fowleri.*** Gene Ontology (GO) graph generated from the cellular component ontology of the proteins, which were up-regulated in highly pathogenic trophozoites compared to weakly pathogenic trophozoites. The specificity of the GO terms increases from the bottom to the top of the graph with arrows indicating “being part of”. The majority of the potential pathogenicity factors localize to the membrane of the cell (blue marked circles), whereas another group of proteins localized to either cell projections (green marked circles) or vesicles (orange marked circles).

Clustering of these newly identified potential pathogenicity factors from *N. fowleri* according to their cellular component GO affiliations suggested localization of the proteins to the cellular membrane, in vesicles and cell projections (Figure 5).

## Discussion

Despite extensive research over the last several decades, the mechanisms accounting for the rapidly progressing and destructive nature of PAM are still unknown. Here, the 30-Mb genome sequence of *N. fowleri*, including RNA sequencing data, is presented for the first time. Based on these data, we identified 22 novel potential pathogenicity factors in the amoeba using proteomic approaches. Moreover, 21 proteins were found to be down-regulated in highly pathogenic trophozoites. Most of this last group of proteins was composed of components of the mitochondrial respiratory chain as well as protein biosynthesis pathways (Table 4). Because they survive at the expense of their host, highly pathogenic *N. fowleri* trophozoites may require a smaller gene repertoire because they may benefit from the host’s cells. In contrast, weakly pathogenic trophozoites are usually free living and therefore must possess all of the genes needed for essential metabolic processes, likely explaining the differences in the metabolism of the highly and weakly pathogenic *N. fowleri*.

**Table 4:** **Differentially expressed proteins in highly and weakly pathogenic** ***N. fowleri,*** **as identified via 1D gel electrophoresis in combination with nano-LC MS/MS**

Accession Swissport	Hit Definition	Species	NP: HP/WP	PLP: HP/WP
Q3SZP7	Villin-1	Bos taurus	16.87	10.19
Q55585	Probable succinate-semialdehyde dehydrogenase [NADP (+)]	Synechocystis sp. PCC 6803 substr. Kazusa	9.09	11.63
Q4UB16	Ras-related protein Rab-1	Theileria annulata	8.88	9.39
P08799	Myosin II heavy chain	Dictyostelium discoideum	8.03	3.86
Q4R550	Cysteine--tRNA ligase, cytoplasmic	Macaca fascicularis	7.35	4.79
B7MMS8	Gamma-aminobutyraldehyde dehydrogenase	Escherichia coli S88	7.17	7.48
O75382	Tripartite motif-containing protein 3	Homo sapiens	6.43	3.91
Q9V4N3	Cytochrome b5; Short = CYTB5	Drosophila melanogaster	4.46	2.62
P27420	Heat shock 70 kDa protein C	Caenorhabditis elegans	4.14	2.77
Q9N1T2	X-linked retinitis pigmentosa GTPase regulator	Canis lupus familiaris	4.12	4.97
D2VAA9	Methylthioribose-1-phosphate isomerase	Naegleria gruberi	3.91	2.90
Q25544	26S protease regulatory subunit 8 homolog	Naegleria fowleri	3.88	2.50
P54772	Histidine decarboxylase	Solanum lycopersicum	2.91	8.22
P10733	Severin	Dictyostelium discoideum	2.88	2.73
Q1HPW4	Eukaryotic translation initiation factor 3 subunit I	Bombyx mori	2.85	2.23
Q54BW4	Circularly permutated Ras protein 2	Dictyostelium discoideum	2.46	3.79
Q12965	Unconventional myosin-Ie	Homo sapiens	2.30	3.56
P34552	Apoptosis-linked gene 2-interacting protein X 1	Caenorhabditis elegans	2.30	2.21
Q54K50	Phospholipase D Y	Dictyostelium discoideum	2.22	2.19
Q5TJ55	Formin-D	Dictyostelium discoideum	2.18	8.77
Q1ZXF7	GDP-mannose 4,6 dehydratase	Dictyostelium discoideum	2.15	2.44
Q8IV36	Protein HID1	Homo sapiens	2.07	2.71
P13629	Periplasmic [Fe] hydrogenase large subunit	Desulfovibrio oxamicus (strain Monticello)	−2.14	−2.86
P08964	Myosin-1	Saccharomyces cerevisiae S288c	−2.25	−5.91
P51824	ADP-ribosylation factor 1	Solanum tuberosum	−2.38	−6.44
A7HBL7	Elongation factor Tu	Anaeromyxobacter sp. Fw109-5	−2.46	−2.42
Q9CR62	Mitochondrial 2-oxoglutarate/malate carrier protein	Mus musculus	−2.56	−3.14
Q1ZXF1	Probable enoyl-CoA hydratase, mitochondrial	Dictyostelium discoideum	−2.69	−4.44
F4P6T0	Ubiquinol oxidase, mitochondrial	Batrachochytrium dendrobatidis JAM81	−2.97	−5.61
P77735	Uncharacterized oxidoreductase YajO	Escherichia coli K-12	−3.06	−2.15
Q889U1	Single-stranded DNA-binding protein	Pseudomonas syringae pv. tomato str. DC3000	−3.19	−3.39
P48375	12 kDa FK506-binding protein	Drosophila melanogaster	−3.55	−2.95
Q2G8K9	Elongation factor Ts	Novosphingobium aromaticivorans DSM 12444	−3.56	−2.27
Q7S6S4	Mitochondrial import inner membrane translocase subunit tim-16	Neurospora crassa OR74A	−3.67	−2.80
P54168	Uncharacterized protein YpgQ	Bacillus subtilis subsp. subtilis str. 168	−3.9	−3.05
O42899	Protein sco1	Schizosaccharomyces pombe 972 h-	−3.91	−7.12
Q97FZ9	Rubrerythrin-1	Clostridium acetobutylicum ATCC 824	−4.94	−2.06
Q9P7M0	ABC1 family protein C21C3.03, mitochondrial	Schizosaccharomyces pombe 972 h-	−5.73	−2.62
P42730	Chaperone protein ClpB1	Arabidopsis thaliana	−6.29	−4.14
P54872	Hydroxymethylglutaryl-CoA synthase A	Dictyostelium discoideum	−6.3	−3.2
Q60597	2-oxoglutarate dehydrogenase, mitochondrial	Mus musculus	−8.13	−2.87
P42125	Enoyl-CoA delta isomerase 1, mitochondrial	Mus musculus	−8.93	−2.44
O67589	Aspartate--tRNA ligase	Aquifex aeolicus VF5	−20.48	−11.26

The proteins are sorted according to their decreasing level of up- or down-regulation in reference to weakly pathogenic *N. fowleri*. The regulation levels are given in columns 4 (NP: HP/WP: highly pathogenic trophozoites in Nelson’s medium versus weakly pathogenic trophozoites in PYNFH medium) and 5 (PLP: HP/WP: highly pathogenic trophozoites in PYNFH/LH medium versus weakly pathogenic trophozoites in PYNFH medium). The 22 up-regulated proteins (HP/WP > 2) were considered to be potential pathogenicity factors of *N. fowleri.*

From a phylogenetic point of view, the *de novo*-assembled *N. fowleri* genome may shed light on the extent of the taxonomic relationship between *N. fowleri* and its non-pathogenic relative *N. gruberi,* whose genome sequence was published in 2010 [8]. To the best of our knowledge, there is no reliable means, at least for eukaryotes, of translating whole-genome-based comparisons into taxonomic relationships. The general approach for assessing phylogenetic relationships is to pick a (set of) gene (s) as the basis for comparison. Using this approach, the level of divergence between *N. fowleri* and *N. gruberi* based on 18S ribosomal DNA analysis has been estimated to be approximately similar to that between mammals and frogs [20]. In a study conducted by Herman *et al.* [9], a lack of collinearity between the *N. fowleri* and *N. gruberi* genomes was found through sequencing a 60-kb nuclear segment from *N. fowleri* and comparing it with corresponding sequences from *N. gruberi*. Furthermore, according to a typing system based on internal transcribed spacers and 5.8S rDNA sequences, there is strong evidence that *Naegleria lovaniensis*, and not *N. gruberi*, is the closest relative of *N. fowleri* [21]. According to the genome similarity network obtained by comparing *N. fowleri* with *N. gruberi, A. castellanii, E. histolytica, T. brucei* and *T. cruzi* based on EST sequences (Figure 2), we found that the extent of the relationship between *N. fowleri* and *N. gruberi* is comparable to that between *T. brucei* and *T. cruzi.* However, as only 32.1% of the assembled *N. fowleri* RNA transcripts aligned to the *N. gruberi* genome in BLASTn searches, we propose that there is low similarity between the coding sequences of *N. gruberi* and *N. fowleri*. In summary, all these findings reflect the intricate phylogeny of the protozoan taxonomy, and our data may add a further piece to this complex puzzle. In the context of our work, clarification of the phylogenetic relationships between *Naegleria* species is critical for choosing an appropriate search strategy for potential pathogenicity factors. Comparative analysis of genomic data from *N. fowleri* and *N. gruberi*, aimed at the identification of the pathogenic mechanisms of *N. fowleri,* has been discussed as a possible option in the field of *Naegleria* research. However, based on the findings described above, this experimental strategy is questionable because the substantial dissimilarities between the genomes of these species may lead to a high number of false positive candidates. In our opinion, the possibility of influencing the pathogenic potential of *N. fowleri* according to the composition of the culture medium is a more promising route for identifying relevant pathogenicity factors. While trophozoites maintained in PYNFH medium showed weak *in vivo* pathogenicity, trophozoites in Nelson’s medium were highly pathogenic in mice. Furthermore, when the PYNFH medium was supplemented with LH, *N. fowleri* trophozoites also converted to the highly pathogenic phenotype [10]. Based on this pathogenicity model, we performed an intra-species comparison of *N. fowleri* using a genomic, transcriptomic and proteomic approach to identify the factors accounting for the pathogenic potential of the amoeba. To exclude proteomic differences caused by the different compositions of the media, we compared both of the highly pathogenic phenotypes (trophozoites in PYNFH/LH medium and trophozoites in Nelson’s medium) with the weakly pathogenic trophozoites (cultivated in PYNFH medium). Among the 950 initially identified ORFs showing one or more peptide matches in the mass spectrometric analysis, only 22 proteins were up-regulated in both comparison groups and were therefore considered potential pathogenicity factors.The pathogenicity of an organism is a complex process and is proposed to result from the interactions of many components, rather than the action of one essential factor. Thus, we clustered the 22 potential pathogenicity factors identified according to their cellular components to determine the compartment with the highest pathogenic activity (Figure 5).

### (Trans-) membrane domain

Based on the GO assignment of the proteins to their cellular locations, the membrane was proposed to be one of the main foci where pathogenic activity occurs. Because adherence of *N. fowleri* to its host cells is a crucial step in inducing a successful infection [22], the membrane (i.e., transmembrane proteins) may play an important role in the pathogenesis of PAM. Based on a previous investigation, a fibronectin-binding protein essential for the interaction of trophozoites with extracellular matrix glycoproteins was identified, suggesting that *N. fowleri* harbors a membrane protein related to the human integrin-like receptor [23]. Furthermore, several studies have shown that *N. fowleri* lyses a wide variety of mammalian target cells *in vitro* through contact-dependent mechanisms [24–26]. This is a further indication of the presence of surface proteins with an essential role in the lytic activity of trophozoites. Lowrey and McLaughlin identified a membrane-associated protein with cytolytic activity against mammalian cells [27]. Another membrane protein, Mp2CL5, was isolated from pathogenic *N. fowleri* and was not found in non-pathogenic *Naegleria* species, suggesting Mp2CL5 as a potential pathogenicity factor [19]. Because Mp2CL was expressed at higher levels in highly pathogenic compared to weakly pathogenic trophozoites, as accessed via comparative 2D gel electrophoresis (Figure 3, Table 3), our analysis confirmed the potential involvement of this membrane protein in the pathogenesis of *N. fowleri*. Thus, we consider Mp2CL5 an important candidate for further examination of its role in the pathogenesis of PAM.

In another study, different membrane-bound glycoproteins involved in resistance to complement-mediated damage were described [28]. Moreover, Fritzinger *et al.* demonstrated the presence of an immunogenic surface protein in *N. fowleri* that was reactive with antibodies to human CD59. Because this CD59-like protein binds complement component C9, it may play a role in resistance to complement lysis. Additionally, it has been shown that the CD59-like protein is shed on membrane vesicles [29]. Generally, *N. fowleri* undergoes membrane vesiculation to remove membrane-deposited C proteins, thereby protecting the amoeba from complement damage [30]. In the present study, we also identified vesicle trafficking as a potential pathogenicity mechanism (see the following section).

### Vesicular trafficking

As mentioned above, *N. fowleri* undergoes membrane vesiculation as a mechanism for resisting complement damage [30]. Because various proteins identified as likely to be involved in the pathogenic mechanisms of *N. fowleri* are stored and ultimately shed in membrane vesicles, vesicular trafficking may play an important role in the pathogenesis of PAM. The CD59-like protein mentioned above is shed in vesicles [29]. Furthermore, the two pore-forming glycoproteins, naegleriapore A and B, are stored in intracellular granular vesicles. As naegleriapore A and B exert cytotoxicity in the form of membrane-permeabilizing activity towards prokaryotic as well as eukaryotic cells, they are proposed to be involved in the pathogenesis of PAM [31]. The vesicular storage of the CD59-like protein, naegleriapores A and B and likely also other potential *N. fowleri* pathogenicity factors may present a means of self-protection from the cytotoxic activity of these factors. Therefore, intracellular vesicles may function as part of a pathogenicity machinery via storing and ultimately secreting proteins that are able to destroy target cells.

Previous studies conducted in our lab have demonstrated the localization of membrane vesicles on highly pathogenic trophozoites maintained in Nelson’s medium, but not on weakly pathogenic trophozoites. However, because no vesicle formation was observed in those trophozoites in PYNFH/LH medium, which were also found to be highly pathogenic, the presence of membrane vesicles could not be related to the *in vivo* pathogenicity [10]. Conversely, based on a combination of findings, the vesicular trafficking system *per se* was characterized as a cellular compartment with potential pathogenic activity (Figure 5). In particular, it was found that apoptosis-linked gene-2-interacting protein X1 (AIP1), which has now been identified as a potential *N. fowleri* pathogenicity factor (Table 4), is a key regulator of endosomal sorting [32]. The endosomal system accomplishes the intracellular transport of cellular material between organelles such as the Golgi apparatus as well as from organelles to the membrane and *vice versa* via vesicles. Yu *et al.* suggested that the Golgi-localized transmembrane protein HID-1, which is up-regulated in highly pathogenic *N. fowleri* (Table 4), may be involved in vesicular exocytosis by preventing the mis-sorting of peptides to lysosomes for degradation [33, 34]. Thus, both AIP1 and HID-1 are interesting candidate *N. fowleri* pathogenicity factors, potentially acting to regulate vesicular trafficking in the amoeba.

In *E. histolytica* it has been shown that, in addition to the storage and secretion of cytolytic molecules (such as amoebapores and cytolytic cysteine proteases), vesicles are implicated in phagocytosis [35–37]. The Rab GTPase EhRabB, which is localized in cytoplasmic vesicles, is involved in the phagocytosis of *E. histolytica* [37, 38]. Rho family GTPases, including Rab proteins such as EhRabB, regulate the cytoskeleton and associated pathogenic processes such as phagocytosis, which in turn, is controlled by vesicular trafficking [39]. Although this topic requires further investigation, the Ras-related protein Rab-1, which was up-regulated in highly pathogenic compared to weakly pathogenic *N. fowleri* in our analysis (Table 4), may be involved in vesicular trafficking and, thus, in the phagocytosis of target cells.

Taking these findings together, vesicular trafficking may be an important step in the pathogenesis of *N. fowleri* infection, as potential pathogenicity factors in the amoeba, including the CD59-like protein and naegleriapores A and B, are stored in vesicles. This possibility is further supported by our analysis showing that vesicular trafficking is regulated by proteins identified as potential pathogenicity factors in *N. fowleri,* such as AIP1, Rab1 and HID-1.

The formation of vesicles via membrane budding involves re-organization of the cytoskeleton, mainly depending on the turnover of actin filaments [30], which is discussed as a potential factor in the pathogenicity of *N. fowleri* in the next section.

### Cell projection

Cell projection was identified as a process that is likely involved in the pathogenesis of PAM (Figure 5). *Naegleria* trophozoites exhibit amoebastomes, or food-cups, which are pseudopodial projections [24, 40]. These amoebastomes are involved in the attachment of amoebae to substrates as well as in the ingestion of bacteria, yeast cells and cellular debris via phagocytic processes [24, 26, 40, 41]. Phagocytosis is dependent on the dynamic turnover of the cytoskeletal protein actin. Because actin is localized around food cups and has been shown to have the capacity to modulate *in vitro* cytotoxicity in different target cells, it is frequently discussed as a potential pathogenicity factor in *N. fowleri* [11–16, 18]. Furthermore, the effects of immunization with either a DNA vaccine or a lentiviral vector expressing the *nfa1* gene (*N. fowleri* actin 1) were investigated in mice infected with PAM [42, 43]. In the present study, actin 1 and actin 2 were found to be up-regulated in highly pathogenic trophozoites in 2D gels (Figure 3, Table 3), confirming the potential role of actin in the pathogenic mechanisms of *N. fowleri.* Another protein showing specific localization around phagocytic food cups that plays a role in cytotoxicity as well as in proliferation of *N. fowleri* is heat shock protein 70 (hsp70) [44]. The potential involvement of hsp70 in the pathogenic mechanisms of the amoeba was confirmed by our analysis detecting the up-regulation of this protein in highly pathogenic trophozoites (Figure 3, Tables 3 and 4).

**Figure 6:**
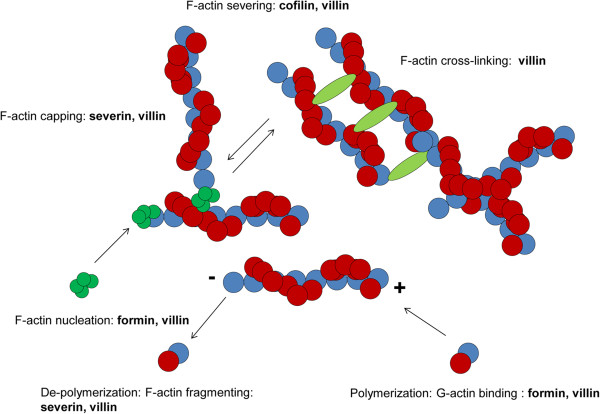
**Regulation of the actin cytoskeleton.** The turnover of actin filaments is strictly regulated by the actin-binding proteins formin, severin, cofilin and villin.

The structural function of the actin cytoskeleton is essential in eukaryotic cells. Actin filaments participate in diverse cellular processes, such as adhesion [45], motility [46] and phagocytosis. The role of actin during phagocytosis has also been reported in other parasites, including *E. histolytica* [47] and *A. castellanii* [48]. Because actin plays a dynamic and structural role in many essential mechanisms in cells, its turnover must be strictly regulated by actin-binding proteins (Figure 6). Formins, such as formin D, which we identified as a potential pathogenicity factor in *N. fowleri* (Table 4), are a crucial class of proteins that regulate the formation of actin filaments. Formins promote the polymerization of monomeric G-actin into filamentous F-actin as well as actin nucleation (Figure 6), ultimately resulting in actin filament assembly through a processive capping mechanism [49, 50]. Another actin-modifying protein with a potential role in the pathogenesis of PAM is severin (Table 4). Severin belongs to the class of actin-fragmenting and -capping proteins (Figure 6) [51]. F-actin fragmentation is required for rapid re-arrangements of the filamentous actin cytoskeleton during cellular processes such as phagocytosis. Cofilin, which showed equal expression levels in weakly and highly pathogenic trophozoites in 2D gels (Figure 3, Table 3), is an actin filament-severing protein that creates free barbed ends that are available for de-polymerization or polymerization (Figure 6) [52, 53]. Finally, villin is a multi-functional actin cytoskeleton-regulating protein that is able to perform all of the actin-modifying functions discussed above, carrying out actin nucleation and polymerization as formin does; actin capping and fragmentation as severin does; actin severing as cofilin does; and actin bundling via cross-linking actin filaments (Figure 6) [54]. In our analysis, villin-1 was the protein found to be up-regulated at the highest level in highly pathogenic *N. fowleri* compared to weakly pathogenic *N. fowleri* (Table 4). Though the exact role of retinitis pigmentosa GTPase regulator (RPGR), which was also identified as a potential pathogenicity factor in *N. fowleri* (Table 4), is unknown, Gakovic *et al.* proposed the involvement of RPGR in the regulation of F-actin [55]. Although this possibility requires further investigation, formin D, severin, villin-1 and RPGR may be involved in actin-dependent pathogenic processes such as phagocytosis. Because of the versatile role of villin-1 in regulating the actin cytoskeleton and the fact that it showed the highest level of up-regulation in highly pathogenic trophozoites, villin-1 is the most promising candidate for further investigations to elucidate the molecular mechanisms involved in the pathogenesis of PAM.

As noted above, phagocytosis is an actin-dependent process. Dianokova *et al.* [56] showed that the actin-binding myosins are concentrated around phagocytic cups in macrophages. Based on the notion that these phagocytic cups are similar to amoebastomes, myosin may be involved in phagocytic processes in the amoeba. We identified myosin II heavy chain as well as myosin Ie as potential pathogenicity factors in *N. fowleri* (Table 4). In macrophages, myosin II is required for the contractile activity of phagocytic cups [57], whereas class I myosins have been proposed to act at the membrane-actin interface to support endocytosis and exocytosis via vesicular trafficking [58]. Thus, further experiments are required to investigate the putative localization of myosin at the site of amoebastomes and to examine its role in the phagocytosis of target cells.

## Conclusions

Using genomic, transcriptomic and proteomic approaches, we identified 22 proteins that potentially act as pathogenicity factors in the deadly amoeba *N. fowleri.* The membrane was identified as a key location where pathogenic processes may occur, and these processes most likely involve actin-dependent vesicular trafficking mechanisms. This study will be the basis for our future application of reverse genetic approaches to demonstrate the role of the identified candidate proteins in the pathogenesis of PAM.

## Methods

### *In vitro*cultivation of *N. fowleri*

Weakly pathogenic *N. fowleri* trophozoites (ATCC 30863) were cultivated at 37°C in 5 ml of buffered PYNFH medium containing 1% (w/v) Bacto™ Peptone (BD Biosciences, Allschwil, Switzerland), 1% (w/v) yeast extract (BD Biosciences), 0.1% (w/v) yeast ribonucleic acid (Sigma, Buchs, Switzerland), 15 mg folic acid (Sigma) l^−1^ and 1 mg hemin (Sigma) l^−1^, supplemented with 10% (v/v) fetal calf serum (in 133 mM KH_2_PO_4_, 176.1 mM Na_2_HPO_4_), in Nunclon^TM^ Δ Surface tubes (Fisher Scientific, Allschwil, Switzerland) from frozen stocks. To generate highly pathogenic *N. fowleri*, trophozoites were transferred either to Nelson’s medium containing 0.1% (w/v) LH (Sigma) and 0.1% (w/v) D-(+)-glucose (Sigma), supplemented with 10% (v/v) fetal calf serum in Page’s amoeba saline (2 mM NaCl, 16 μM MgSO_4_, 27.2 μM CaCl_2_, 1 mM Na_2_HPO_4_, 1 mM KH_2_PO_4_), or to PYNFH medium supplemented with 0.1% (w/v) LH [10].

### Genomic DNA sequencing

#### DNA isolation and library preparation

DNA was extracted from 10^8^ *N. fowleri* trophozoites cultivated in Nelson’s medium using the DNeasy Blood and Tissue Kit (Qiagen, Basel, Switzerland) according to the manufacturer’s protocol. To obtain RNA-free DNA, RNA digestion was performed using 4 μl of RNase A (Qiagen). The DNA was eluted with 100 μl of 10 mM TrisHCl, pH 8.5, pre-heated to 70°C. The DNA quality was visualized on 0.8% agarose gels, and quantification was performed using a NanoDrop® device. Three micrograms of high-molecular weight DNA was sent to Fasteris (Plan-les-Quates, Switzerland) for paired-end sequencing, with an insert size of 300 bp, using the Illumina HiSeq 2000 platform, while 20 μg of DNA was sent to GATC Biotech (Constance, Germany) for preparation of a 3-kb mate-pair library using Illumina technologies and for Roche 454 GS FLX sequencing.

The NGS reads have been deposited in DDBJ/EMBL/GenBank under accession SRX523949 (Illumina HiSeq 2000 reads) and SRX547942 (Roche 454 GS FLX reads).

#### PCR analysis of the contents of ribosomal and mitochondrial DNA relative to the genomic DNA

In addition to its nuclear genome, *N. fowleri* has multiple copies of an extrachromosomal plasmid encoding ribosomal DNA [59, 60] and a 50-kb mitochondrial genome [9]. To avoid bias in the contents of ribosomal and mitochondrial DNA relative to genomic DNA, DNA extracted for whole-genome sequencing was subjected to PCR analysis specifically targeting 18S rDNA, mitochondrial DNA and a glutathione S transferase III homolog (EMBL:U43126) in the genome. The primers used for PCR are listened in the Additional file 1.

#### Genome size and ploidy level estimation via flow cytometry

Flow cytometry is a method that is widely used to measure genome sizes in plants [61]. We estimated the genome size and level of ploidy of *N. fowleri* based on the known genome sizes of *Giardia lamblia* and *Trichomonas vaginalis*. Pellets of approximately 10^7^ *N. fowleri, G. lamblia* and *T. vaginalis* were diluted in PBS to a concentration of 10^5^ trophozoites/ml. To stain the nuclei, 20 μl of SYBR Green I (Invitrogen, Lucerne, Switzerland), which intercalates into the DNA [62], was added to the samples, followed by incubation for 20 minutes in the dark. Flow cytometric detection was performed with a Partec CyFlow® SL flow cytometer (Partec GmbH, Münster, Germany) equipped with a 488-nm blue solid-state laser operating at 20 mW. The trigger was set to green fluorescence. The flow speed rate was 3 μl/second, implying a counting rate of less than 10^3^ events/second. The results were acquired using Partec FlowMax software, v2.4d. The calculation of the genome size of *N. fowleri* was based on the known genome sizes of the reference species (e.g., 48 Mb for *G. lamblia* [63] and 177 Mb for *T. vaginalis* [64]) and the relative green fluorescence in the co-prepared samples [65].

### *De novo*assembly

Because no reference genome exists for *N. fowleri*, the sequencing reads were assembled *de novo* using CLC 4.7.1. Raw reads were trimmed for removal of low-quality sequences (with a limit of 0.05) and for ambiguous nucleotides. The Roche 454 read ends were additionally screened and trimmed for 454 adapter sequences. Genome assembly was performed with the CLC *de novo* sequencing tool using the default parameters, with a minimum contig length of 800 bp and with scaffolding. CLC assembly is a two-step process based on the De Bruijn graph algorithm. First, contig sequences are built based on information included in the read sequences, such as paired-end information. In a second step, to show the coverage levels, all reads are mapped back using the contig sequences obtained as a reference.

The results of this whole-genome shotgun project have been deposited in DDBJ/EMBL/GenBank under accession AWXF00000000 and in the eukaryotic pathogen database EuPathDB (http://eupathdb.org/eupathdb/). The version described in this paper is version AWXF01000000.

### RNA sequencing

#### RNA isolation and library preparation

RNA was extracted from 10^7^ *N. fowleri* trophozoites cultivated in Nelson’s medium using the EZ1 RNA Universal Tissue Kit (Qiagen) and the EZ1 BioRobot (Qiagen). Trophozoites were resuspended in 750 μl of QIAzol lysis reagent (Qiagen), followed by disruption and homogenization by operating a TissueLyser at 25 Hz for 3 min. After incubation for 5 min at room temperature, 150 μl chloroform (Grogg, Stettlen, Switzerland) was added to the homogenized samples. The mixture was then centrifuged for 15 min at 12,000 g at 4°C, and the upper aqueous phase was used as the starting material for RNA isolation with the EZ1 BioRobot, according to manufacturer’s protocol. Quantification and examination of the total RNA integrity was performed with the Agilent 2100 Bioanalyzer system. Four micrograms of high-quality RNA was sent to the Next Generation Sequencing Platform of the University of Bern for paired-end sequencing by the Illumina HiSeq 2000 device.

The reads from RNA sequencing have been deposited in DDBJ/EMBL/GenBank under accession SRX553040.

#### *De novo*assembly and ORF prediction

To obtain high-quality transcriptome sequence data, raw reads were trimmed via the removal of low-quality sequences (with a limit of 0.05) and based on ambiguous nucleotides. The trimmed reads were then *de novo-*assembled into transcripts with Trinity, a three-module software pipeline specifically developed for *de novo* transcriptome assembly [66]. Trinity generates contigs, clusters the contigs into individual groups, with each representing the full transcriptional complexity of a given gene, and then constructs a De Brujin graph for each contig group. To identify ORFs, protein-coding regions were extracted from Trinity transcripts by a downstream application of the program. High redundant ORFs were filtered over a 95% identity threshold using the program cd-hit (http://weizhong-lab.ucsd.edu/cd-hit/). The resulting ORFs were used as a database for protein identification via nano-LC MS/MS (see below). To assess the accuracy of the assembled transcripts, each transcript was aligned to our genomic data using the CLC Mapping tool with the default parameters.

#### Genome similarity of *N. fowleri*and *N. gruberi*

To compare the genetic diversity of the genome of *N. fowleri* with its non-pathogenic relative *N. gruberi* and with the more distantly related species *A. castellanii, E. histolytica, T. brucei* and *T. cruzi*, Evolutionary Gene and Genome Network (EGN) software was used [67]. EGN generates genome networks from molecular datasets by comparing sequences via BLAST homology searches. As input files for comparison with our gene-finding data, EST sequences from *N. gruberi, A. castellanii, E. histolytica, T. brucei and T. cruzi* were downloaded from the National Center for Biotechnology Information website (NCBI, http://www.ncbi.nlm.nih.gov/). The genome network was generated at an e-value cutoff of 3 and a 20% identity threshold. The EGN output file was imported into Cytoscape 3.0.1 [68] to visualize the genome network as a graph, with nodes representing the organisms and edges representing the similarity between two nodes (Figure 2). The length of an edge is represented as the inverse proportion of shared gene families.

Moreover, the 17,252 predicted ORFs from *N. fowleri* were queried against the genome of *N. gruberi* (NCBI:ACER00000000.1) as well as against the *de novo*-sequenced genome of *N. fowleri* using BLASTn [69]. The applied parameters were as follows: match = 2, mismatch = −3, gap costs for existence = 5 and for extension = 2. The minimal hit length was set to 100 nucleotides.

For standalone BLASTp protein comparison against the RefSeq database, default parameters were applied. The resulting XML file was then used for functional annotation by the CLC plugin Blast2GO.

### Proteomics

#### 2D gel electrophoresis

To identify potential pathogenicity factors in *N. fowleri,* the proteomes of weakly and highly pathogenic trophozoites were separated via 2D gel electrophoresis, and differing protein spots were analyzed through nano-LC MS/MS. Pellets of 10^7^ trophozoites were washed 3 times in PBS, and 10 μl of the Halt Protease Inhibitor Single-Use Cocktail (Thermo Scientific) was added. Cell disruption was performed through 3 cycles of freezing (liquid nitrogen) and thawing, followed by re-solubilization in 7 M urea, 2 M thiourea, 1% DTT and 4% CHAPS containing 0.5% ampholytes, pH 5–8 (Bio-Rad, Cressier, Switzerland), operating a Bioruptor® UCD-200 for 15 min at high intensity. Twenty-five micrograms of protein (determined by the Bradford Assay) was applied to an IEF strip (Bio-Rad) via in-gel re-hydration for 12 h at 50 mV, after which isoelectric focusing (IEF) was performed for a total of 32 kVh. After IEF, the strips were reduced in equilibration buffer (6 M urea, 50 mM Tris pH 8.8, 2% SDS, 30% glycerol) containing 1% DTT for 10 min, followed by alkylation in equilibration buffer containing 4% iodoacetamide for 10 min. The second dimension was run on a precast 4-15% gradient polyacrylamide gel (Bio-Rad) at a constant voltage of 200 V. The separated proteins were visualized using the SilverQuest™ Silver Staining Kit (Invitrogen) according to the manufacturer’s protocol. Protein spots to be analyzed by nano-LC MS/MS were excised and destained with 50 μl of Destainer A and 50 μl of Destainer B (SilverQuest™ Silver Staining Kit). For each condition, spots from three 2D gels were analyzed.

#### 1D gel electrophoresis

To obtain an additional, broader overview of the *N. fowleri* proteome, proteins from weakly as well as highly pathogenic trophozoites were separated by 1D gel electrophoresis and identified through nano-LC MS/MS. Pellets of 10^7^ trophozoites were resolubilized for 3 min via sonication in a water bath with 5 mM HEPES, pH 7.4, and 50 mM mannitol containing a protease inhibitor cocktail (Roche). Protein concentrations were determined by OD280 nm measurement, and aliquots corresponding to 30 μg of protein were separated by SDS-PAGE (10%). Each sample lane was cut into 27 slices from top to bottom for in-gel digestion and nano-LC MS/MS.

#### Nano-LC MS/MS

For protein identification, protein spots excised following 2D gel electrophoresis and slices excised following 1D gel electrophoresis were further processed for nano-LC MS/MS analysis. The 2D gel spots were digested directly, while the gel slices were reduced and alkylated prior to digestion with trypsin and analyzed by nano-LC MS/MS as described in [70]. The generated fragment spectra were searched against our personal ORF database obtained from RNA sequencing (see above) using EasyProt software [71].

#### Data mining and annotation

Based on data from 1D gel electrophoresis, in combination with nano-LC MS/MS, differentially expressed proteins were identified by summing all of the scores from peptide spectral matches to one particular ORF, which is termed protein match score summation (PMSS) [72], and calculation of the relationship of the PMSS of highly pathogenic to weakly pathogenic *N. fowleri.* Then, ORFs with a relative PMSS value equal to or greater than 2, or equal to or less than −2 were considered to be differentially expressed proteins between the two conditions.

For annotation, the differentially expressed ORFs were subjected to searches with the next-generation sequence similarity search tool ngKLAST (http://www.korilog.com). A KLASTp search was run against the annotated Swissprot database under default settings. KLAST hits with a bit score greater than or equal to 50 were considered significant and were used for further analysis.

Data clustering based on GO terms was carried out on the R platform for statistical programming using packages from the Bioconductor project [73]. To retrieve GO identifiers associated with the Uniprot Accession numbers of significant KLAST hits, the biomaRt package was used, which implements the BioMart software suite [74, 75].

## Electronic supplementary material

Additional file 1: **Primer sequences.** Primers used to determine the contents of ribosomal and mitochondrial DNA relative to the genomic DNA used as the starting material for genome sequencing. (XLSX 12 KB)

## Abbreviations

AIP1: Apoptosis-linked gene-2-interacting protein X1
CLC: CLC Genomic Workbench
EGN: Evolutionary Gene and Genome Network
GO: Gene Ontology
Hsp70: Heat shock protein 70
IEF: Isoelectric focussing
LH: Liver hydrolysate
Nano-LC MS/MS: Nano-liquid chromatography tandem mass spectrometry
NCBI: National Center for Biotechnology Information
ORFs: Open reading frames
PAM: Primary amoebic meningoencephalitis
PMSS: Protein match score summation
RPGR: Retinitis pigmentosa GTPase regulator.
